# *In Vivo* Effects of Pichia Pastoris-Expressed Antimicrobial Peptide Hepcidin on the Community Composition and Metabolism Gut Microbiota of Rats

**DOI:** 10.1371/journal.pone.0164771

**Published:** 2016-10-21

**Authors:** Lanfang Tian, Siyuan Chen, Haiyan Liu, Mingzhang Guo, Wentao Xu, Xiaoyun He, Yunbo Luo, Xiaozhe Qi, Hongxia Luo, Kunlun Huang

**Affiliations:** 1 Beijing Advanced Innovation Center for Food Nutrition and Human Health, College of Food Science and Nutritional Engineering, China Agricultural University, Beijing, 100083, PR China; 2 Beijing Vocational College of Agriculture, Beijing, 102442, PR China; 3 School of Public Health, North China University of Science and Technology, Tangshan, 063000, Hebei, PR China; 4 Beijing Laboratory for Food Quality and Safety, Beijing, 100083, PR China; Wageningen University, NETHERLANDS

## Abstract

Hepcidin, one kind of antimicrobial peptides, is one of the promising alternatives to antibiotics with broad spectrum of antimicrobial activity. Hepcidins cloned from different kinds of fishes have been produced using exogenous expression systems, and their *in vitro* antimicrobial effects have been verified. However their *in vivo* effects on gut microbiota and gut health of hosts remain unclear. Here we performed a safety study of hepcidin so that it can be used to reduce microbial contaminations in the food and feed. In this study, *Pichia pastoris*-expressed *Pseudosciaena crocea* hepcidin (PC-hepc) was first assessed by simulated digestion tests and then administered to male and female Sprague-Dawley (SD) rats in different concentrations. Subchronic toxicity testing, high throughput 16S rRNA sequencing of gut microbiota, and examinations on gut metabolism and permeability were conducted. The results showed PC-hepc could be digested in simulated intestinal fluid but not in simulated gastric fluid. PC-hepc had no adverse effects on general health, except causing increase of blood glucose (still in the normal value range of this index) in all trial groups of female rats and intestinal inflammation in HD group of female rats. Community composition of gut microbiota of female MD and HD groups shifted compared with control group, of which the decrease of genus *Akkermansia* might be related to the increase of blood glucose and intestinal inflammation. Significant increase of fecal nitroreductase activity was also observed in female MD and HD groups. Our results suggest the uses of exogenous PC-hepc in normal dosage are safe, however excess dosage of it may cause intestinal disorder of animals.

## Introduction

Bacterial disease outbreaks are great threats to human health and agricultural industries. To prevent or treat bacterial diseases, antibiotics have been widely used. However, several mechanisms have made bacterial pathogens either to be intrinsically resistant or to acquire resistance to certain antibiotics [1], which results in the emergence of multidrug-resistant strains among major Gram-positive and Gram-negative species [2], causing huge economic losses and public health crisis. Another concern regarding the use of antibiotics is their effects on the gut microbiota of animals. In recent years, new sequencing technologies have enabled the study of microbiota response to antibiotic disturbance and have shown that antibiotics do not simply impact one potential pathogen, but also change the ecology within the gut. The challenges of antibiotics to the gut microbiota include alteration in bacterial membership [3] and reduction of the immune responses in peripheral organs [4]. With these crises, there is an urgent need for the development and validation of alternatives to antibiotics.

Antimicrobial peptides (AMPs) represent one of the most promising strategies for substituting antibiotics. They are small, positively charged, amphipathic molecules of variable amino acid composition and length [5]. Approximately 1500 kinds of AMPs have been characterized in a wide range of organisms, both prokaryotes and eukaryotes [6]. It is believed that these peptides function as broad-spectrum microbicides and form part of the innate immune system of many eukaryotes, including humans.

Hepcidin was one kind of the most studied AMP families in the last 20 years. Hepcidins have been identified in mammals [7] and fishes [8]. The sequence conservation between hepcidins from mammals and fishes is remarkably high [9]. In mammals, hepcidins are reported to be associated with antimicrobial activity and iron metabolism [10]. In fishes, hepcidin gene expression is mostly associated with bacterial infection or inflammation [11], with only very few studies indicating that hepcidins function as iron regulators in fishes [12]. The in vitro antimicrobial activity of fish hepcidin has been studied. Most of these hepcidins exhibited antibacterial or antifungal effects, although the spectrum was variable [11, 13, 14]. These in vitro tests indicate that hepcidins are promising microbicides or feed additives in breeding industry.

Due to the promising application, many kinds of hepcidins have been produced using exogenous expression system. However, two concerns need to be addressed prior to the practical application of hepcidins: first, as genetic engineering peptides as well as homologues to endogenous hepcidins of human and animals, whether exogenous hepcidins have adverse effects on health of human or animals; second, whether hepcidins can affect the composition or function of gut microbiota in the same manner as antibiotics. No research on the safety assessment of oral intake of exogenous hepcidin has been reported. In this study, safety study of *Pichia pastoris*-expressed large yellow croaker (*Pseudosciaena crocea*) hepcidin (PC-hepc) was conducted using Sprague-Dawley (SD) rats in different concentration, and the above concerns were examined through subchronic toxicity testing and high throughput 16S rRNA sequencing of gut microbiota. The results showed low dose (LD, 0.01 g/kg) of PC-hepc was safe for rats by oral intake. However, high dose (HD, 0.5 g/kg) of PC-hepc caused jejunum inflammation and change of gut microbiota in female rats. Medium dose (MD, 0.1 g/kg) of PC-hepc caused similar changes of gut microbiota to those observed in HD group in female rats, but didn’t cause inflammation.

## Materials and Methods

### Hepcidin Samples

*Pichia pastoris*-expressed PC-hepc was provided by State Key Laboratory of Marine Environmental Science, College of Oceanography and Environmental Science, Xiamen University. The PC-hepc gene was cloned from *Pseudosciaena crocea* and expressed by *Pichia pastoris*. Purified peptides were freeze-dried into powders and then stored at −20°C until use.

### In vitro simulated digestion

The PC-hepc was digested in simulated gastric fluid (SGF) and simulated intestinal fluid (SIF) as previously described [15]. Briefly, 100 μl of PC-hepc peptides (5 mg/ml for SGF, 2 mg/ml for SIF) were added to 1.9 ml SGF or SIF (Sigma-Aldrich, St Louis, USA). The mixture was incubated at 37°C for 0 s, 15 s, 2 min, 30 min and 60 min, and then the samples were taken out and SDS-PAGE was performed.

### Subchronic Toxicity Testing

Animal experiments were conducted at the Specific Pathogen Free (SPF) animal laboratory of the Supervision and Testing Center for GMO Food Safety, Ministry of Agriculture (Beijing, China; license number SYXK (Beijing) 2010–0036). All procedures were performed in strict accordance with the EU Directive 2010/63/EU for animal experiments and regulations of the Ethics Committee of China Agricultural University. The Ethics Committee of China Agricultural University approved the total procedures and potential mortality aspects of this study with the approval license number JY140102. Four-week-old SD rats (40 males and 40 females) were purchased from Vital River Laboratories, Inc. (Beijing, China) with mean body weight of 100 g. Groups of five rats shared each stainless steel with *ad libitum* access to water and fodder. The animal room was maintained at a temperature of 22 ± 2°C, relatively humidity of 40–70%, and artificially illuminated with fluorescent lights in a 12 h light/dark cycle. Animals were acclimatized for 5 days before being randomly divided into 4 groups, with each group consisting of 10 female and 10 male rats. The negative control group (CK group) was fed with the normal chow diet. The treated groups were fed with diets formulated with 0.01 g/kg (LD group), 0.1 g/kg (MD group), and 0.5 g/kg (HD group) of hepcidin. All diets were manufactured by KeAoXieLi Feed Co Ltd (Beijing, China). All ingredients were adjusted to meet the requirement of rats (GB 14924.3–2010) and exposed to Co60 to induce sterility.

Animals were observed once a day throughout the trial duration for mortality and signs of toxicity. Body weight and food consumption were recorded once a week. No animals died without euthanasia during the intervention. The substance we studied is one kind of antimicrobial peptides, without known toxicity report before our study. During the study, no adverse effect was observed on the general health of any rats. The blood glucose increased in female rats, but we only known this effect at the end of experiment when the serum chemistry test was conducted. Thus no euthanasia was used before the end of experiments.

At the end of the experiment (90 days), total body weight and total food consumption were tallied. Mean food consumption was calculated according to the following formula: mean food utilization (%) = (total body weight gain / total food consumption) × 100.

All rats were fasted overnight before blood sampling. Blood was collected from the orbital venous plexus of the rats on day 90. Blood was stabilized by EDTA (ethylenediamine tetra-acetic acid) and was analyzed by a HEMAVET 950FS animal blood cell counter (Drew Scientific, Dallas, USA). Serum was acquired by centrifugation, and routine serum chemistry parameters were measured by an automatic Biochemical Analyzer 7020 (HITACHI, Tokyo, Japan).

On day 90, rats were anesthetized using chloral hydrate (6% wt/vol, 5 ml/kg) and decapitated. A complete gross necropsy was conducted on all animals. The following organs were weighed: the brain, heart, lungs, thymus, adrenals, kidneys, spleen, liver and testes or ovaries. Tissue sections were fixed in 10% neutral buffered formalin. Fixed tissues were embedded in paraffin and stained by hematoxylin and eosin for microscopic examination. Histopathological examination of tissue sections was conducted at the Experimental Animal Research Center, China Agricultural University.

### Gut Microbiota and Gut Healthy Analysis

Feces from each rat was collected on day 0 and day 90 and stored at −80°C until use. Microbial genomic DNA was extracted from each fecal sample (0.2 g) by a previously described method [16]. The V4 region of the 16S rRNA was amplified by PCR and sequenced by a MiSeq platform (Illumina, San Diego, USA). After data filtering, operational taxonomic unit (OTU)-based taxonomy cluster was performed at 97% identity using QIIME pipeline [17], and taxonomy annotation was conducted using RDP classifier [18] and GreenGene database [19]. The α diversity of each group was calculated based on OTU data using diversity/diversity-indices and diversity/beta-diversity of the software PAST version 2.17 [20]. ANOSIM was performed based on OTU data using multivar/one-way-ANOSIM of PAST with the distance measure set as Bray-Curtis. To find key genus profiles to distinguish between the treated and untreated groups, LEfSe analysis was performed using the default parameters [21].

For total bacteria quantification measurement, the SYBR Green I dye based real-time PCR was performed. Genomic DNA extracted from the fecal samples was used as the templates. 338F: ACTCCTACGGGAGGCAGCAG and 518R: ATTACCGCGGCTGCTGG were used as primers. Genomic DNA of *Escherichia coli* with a detection range from 10^3^ to 10^12^ DNA copies/μl was used to create a standard curve [22].

Determination of fecal short-chain fatty acid (SCFA), analysis of fecal enzyme activity and intestinal permeability was conducted as previously described [22]. Acetic acid, propionic acid and butyric acid were determined by gas chromatography (Agilent 7980N, Santa Clara, USA). Approximately 0.05 g of feces was weighed and suspended in 0.5 ml 0.9% NaCl, homogenized and stored at −20°C overnight. Then, 0.2 ml of 50% H2SO4 was added to each mixture and centrifuged at 12000 r for 15 min. The supernatant was centrifuged again and filtered using a 2 μm diameter filter membrane. The extracts were added to 20 μl of 2-methyl valerate and 600 μl of diethyl ether. One microliter of the extracts from the feces was injected into the gas-liquid chromatography (Hewlett Packard 5890 Series II with a flame ionization detector, Palo Alto, USA). Pure acetic acid, propionic acid and butyric acid (Sigma Chemical, St. Louis, USA) were used to make a standard curve for the calculations. The gas chromatography settings were as follows: an initial oven temperature at 40°C for 2 min, then increased at 4°C /min to 160°C, and retained for 2 min, then increased at 20°C /min to 230°C and retained for 5 min. The detector temperature was set at 230°C. Split ratio was 30:1, rate of flow was 1.2 ml/min, N2 40 ml/min, H2 40 ml/min, combustion air 400 ml/min, and the temperature of the inject port was 180°C.

### *In vitro* inhibition assay of undigested and digested PC-hepc on gut microbial strains

The antimicrobial activity of the undigested and digested PC-hepc was determined against gut microbial Gram-positive strains *Lactobacillus plantarum*, *Lactobacillus rhamnosus*, *Enterococcus faecalis*, *Pediococcus pentosaceus*, *Brevibacterium* spp., and Gram-negative *Escherichia coli*.

The *in vitro* effects of undigested and digested PC-hepc on gut microbial strains were determined according to the method described by Wang et al. (14). Briefly, bacteria were cultured to logarithmic phase and were suspended in fresh broth to a final concentration of about 10^6^. 50 μl of bacterial suspension were mixed with 50 μl the following solution respectively a) 0 μM PC-hepc, b) 3 μM PC-hepc, c) 12μM PC-hepc, d) 48 μM PC-hepc, e) 0 μM digested PC-hepc f) 3 μM digested PC-hepc, g) 12 μM digested PC-hepc, and g) 48 μM digested PC-hepc. All the PC-hepc were dissolved in the PBS buffer, and digested PC-hepc were prepared using the same simulated digestion system as described in section 2.2. After 24 h of incubation at 28°C, the mixture samples were plated on LB agar plates and incubated at 37°C for 24 h for colony counting. Each assay was repeated three times independently.

### Statistical analysis

All values in the Tables 1–4 were expressed as arithmetic means ± standard deviation (s.d.). Data of subchronic toxicity testing and total bacteria quantification, short-chain fatty acid measurement, fecal enzyme activity and intestinal permeability assessment were analyzed using one-way analysis of variance (ANOVA) followed by t-test. A Bonferroni adjustment was not used in the comparison because of excessive reduction in power of tests.

**Table 1:** Final body weight, total food consumption and food utilization.

	Hepcidin content (g/kg)	Final body weight (g)	Total weight gain(g)	gain(g)consumption(g)	Food utilization (%)
Male					
CK	0	544.9±57.8	418.5±58.83	2472.63±98.3	16.93±2.38
LD	0.01	541.8±57.4	414.7±59.48	2295.56±17.25	18.07±2.59
MD	0.1	543.3±48.0	423.9±44.16	2487.32±3.48	17.04±1.78
HD	0.5	534.4±55.4	415.0±53.72	2485.26±161.14	16.7±2.16
Female					
CK	0	302.8±29.0	199.7±26.24	2070.9±304.51	9.64±1.27
LD	0.01	295.3±22.4	192.3±19.75	1898.68±198.16	10.13±1.04
MD	0.1	288.6±22.1	182.4±20.91	1851.51±122.15	9.85±1.13
HD	0.5	301.1±32.1	198.1±30.50	1751.54±102.47	11.31±1.74*

There were ten rats per group. Values are expressed as the means ± standard deviation. The superscript

* indicates significant difference compared with CK group (p<0.05). Food utilization was calculated as total body weight gain / total food consumption.

**Table 2:** Terminal blood hematology of rats fed with PC-hepc for 90 days.

		CK		LD		MD		HD	
**Males**									
WBC (10^9^/L)		11.52±3.71		8.29±2.16*		8.71±2.56		9.7±2.16	
RBC (10^12^/L)		12.1±1.38		12.36±1.49		12.36±2.21		11.61±1.49	
Hb (g/dL)		161.83±7.53		169.33±5.5*		170.83±9.22		165±9.43	
MCH (pg)		13.56±1.56		13.88±1.63		14.15±2.23		14.36±1.43	
RDW (fl)		47.65±5.01		42.83±7.87		34.77±15.1*		49.15±1.66	
PLT (10^9^/L)		1256.17±149.94		1306.89±132.85		1291.5±199.7		1165.19±328.97	
MPV (%)		6.78±0.23		6.56±0.24		6.83±0.41		6.67±0.43	
Ne%		28.9±5.7		34.5±5.6		39.1±3.3*		30.6±4.2	
Ly%		61.2±5.8		55.3±5.9*		53.6±4*		60.3±6	
Eo%		2.17±1.15		2.01±0.99		2.04±1.08		1.89±1.29	
Mo%		7.17±1.39		7.81±1.88		4.94±1.18		7.12±2.85	
**Females**									
WBC (10^9^/L)		7.21±3.16		9.59±2.4		7.48±1.92		7.48±1.92	
RBC (10^12^/L)		11.88±2.01		11.5±1.19		11.85±1.13		11.85±1.13	
Hb (g/dL)		160.33±19.11		155.25±6.61		155.38±13.39		155.38±13.39	
MCH (pg)		13.68±1.91		13.6±1.1		13.16±1.17		13.16±1.17	
RDW (fl)		27.12±14.73		40.46±9.96		35.4±15.33		35.4±15.33	
PLT (10^9^/L)		1252±179		1261±179		1042±296		1042±296	
MPV (%)		6.71±0.57		6.41±0.37		6.66±0.5		6.66±0.5	
Ne%		32.5±6.9		26.7±6.6		27.9±2.4		31.9±8.3	
Ly%		58.5±8.1		63.6±7.4		60±3.8		57.8±10	
Eo%		1.87±1.85		3.29±2.12		3.3±1.79		2.2±2.33	
Mo%		6.54±1.78		5.34±0.98		7.28±1.95		7.28±1.95	

There were ten rats per group. Values are expressed as the means ± standard deviation. The superscript

* indicates significant difference compared with CK group (p<0.05). WBC, white blood cell; RBC, red blood cell; Hb, hemoglobin; MCH, mean corpuscular hemoglobin concentration; RDW, red blood cell distribution width; PLT, blood platelet; MPV, mean platelet volum; Ne%, percentage of neutrophils; Ly%, percentage of lymphocyte; Eo%, percentage of eosinophil; Mo%, percentage of monocytes.

**Table 3:** Terminal serum chemistry of rats fed with PC-hepc for 90 days.

	CK	LD	MD	HD
Male				
ALT (U/L)	44.00±13.86	47.71±17.57	46.00±21.33	44.50±12.66
AST (U/L)	270.25±52.38	275.29±36.94	248.88±93.76	253.30±32.60
TP (g/L)	64.55±6.43	70.56±5.44	67.23±4.41	64.61±2.99
ALB (g/L)	29.00±1.62	33.26±4.43*	31.50±2.93	29.60±1.53
ALP (U/L)	104.50±31.25	89.57±34.70	64.00±21.55*	83.60±31.67
Glu (mmol/L)	6.23±1.96	4.94±1.84	4.84±1.82	6.28±3.47
BUN (mmol/L)	9.15±0.74	8.96±0.69	7.49±2.9	8.93±0.77
Crea (μmol/L)	12.49±1.70	12.37±2.28	11.96±2.19	13.18±3.32
Ca (mmol/L)	2.23±0.08	2.26±0.08	2.24±0.07	2.23±0.04
P (mg/dL)	4.31±0.66	4.21±1.11	3.67±0.86	4.35±1.12
CHO (mg/dL)	1.79±0.48	2.12±0.29	1.96±0.53	1.79±0.40
TG (mmol/L)	0.46±0.10	0.43±0.17	0.44±0.21	0.47±0.23
LDH (U/L)	2675±641	2758±575	2131±449	2394±855
CL (mmol/L)	102.55±1.42	101.73±2.36	102.43±1.41	103.08±2.31
Female				
ALT (U/L)	47.00±9.31	37.00±14.81	45.00±9.00	47.44±12.40
AST (U/L)	264.29±35.42	242.63±78.96	218.22±58.86	294.67±45.32
TP (g/L)	69.01±8.89	62.84±6.31	60.88±3.19*	65.60±5.91
ALB (g/L)	35.24±5.67	30.56±4.34	30.71±2.37	32.61±2.88
ALP (U/L)	65.57±44.11	101.75±63.39	71.56±52.11	66.78±55.48
Glu (mmol/L)	4.16±1.68	6.12±1.65*	6.28±1.85*	6.59±2.01*
BUN (mmol/L)	9.16±1.03	9.64±2.68	9.78±1.98	9.16±1.15
Crea (μmol/L)	12.43±2.29	12.03±2.00	11.17±2.13	14.08±2.82
Ca (mmol/L)	2.33±0.15	2.19±0.19	2.20±0.08	2.31±0.10
P (mg/dL)	4.16±1.19	3.04±0.37*	3.21±0.72	3.69±0.72
CHO (mg/dL)	1.95±0.40	1.5±0.36*	1.54±0.38	1.93±0.63
TG (mmol/L)	0.51±0.33	0.30±0.14	0.30±0.16	0.46±0.25
LDH (U/L)	2300±684	2401±1166	1771±719	1999±565
CL (mmol/L)	102.87±0.98	101.98±4.14	102.00±1.99	102.87±0.87

There were ten rats per group. Values are expressed as the means ± standard deviation. The superscript

* indicates significant difference compared with CK group (p<0.05). ALT, alanine aminotransferase; AST, aspartate transaminase; TP, total phosphorus; ALB, albumin; ALP, alkaline phosphatase; Glu, glucose; BUN, blood urea nitrogen; Crea, creatinine; Ca, CalciumshotNmmdia; P, phosphorus; CHO, cholesterol; TG, triglyceride; LDH, lactate dehydrogenase; CL, chlorine.

**Table 4:** Organ/body weight ratio (%) of male and female rats on the day 90.

	CK	LD	MD	HD
Male				
Brain	0.38±0.05	0.39±0.04	0.38±0.03	0.40±0.03
Liver	2.35±0.17	2.33±0.08	2.77±0.72	2.31±0.17
Spleen	0.14±0.03	0.15±0.03	0.15±0.03	0.15±0.03
Heart	0.33±0.02	0.33±0.03	0.35±0.04	0.35±0.04
Lungs	0.35±0.06	0.33±0.02	0.40±0.10	0.35±0.02
Thymus	0.09±0.02	0.10±0.05	0.09±0.02	0.08±0.01
Kidneys	0.64±0.06	0.60±0.04	0.69±0.13	0.69±0.07
Adrenals	0.015±0.003	0.015±0.003	0.016±0.003	0.016±0.004
Testes	0.63±0.10	0.64±0.12	0.63±0.06	0.63±0.08
Female				
Brain	0.64±0.08	0.65±0.07	0.67±0.06	0.59±0.1
Liver	2.60±0.18	2.84±0.62	2.63±0.14	2.63±0.20
Spleen	0.16±0.01	0.17±0.02	0.17±0.03	0.18±0.02
Heart	0.39±0.05	0.37±0.06	0.37±0.02	0.39±0.07
Lungs	0.48±0.09	0.49±0.07	0.50±0.04	0.51±0.03
Thymus	0.11±0.01	0.11±0.03	0.11±0.03	0.12±0.03
Kidneys	0.66±0.03	0.67±0.08	0.69±0.06	0.71±0.06*
Adrenals	0.035±0.009	0.032±0.008	0.034±0.006	0.033±0.007
Ovaries	0.07±0.03	0.07±0.02	0.07±0.01	0.07±0.02

There were ten rats per group. Values are expressed as the means ± standard deviation. The superscript

* indicates significant difference compared with CK group (p<0.05).

## Results

### PC-hepc could be digested in simulated intestinal fluid but not in simulated gastric fluid

**Fig 1:**
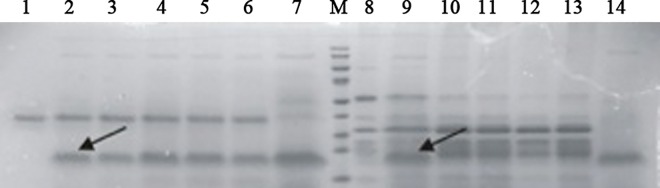
SDS-Page of the digestion products of PC-hepc by simulated gastric fluid and simulated intestinal fluid. Lane 1–7 shows simulated digestion by gastric fluid and Lane 8–14 shows simulated digestion by intestinal fluid. Lane 1 and 8 show digestive enzyme control. Lane 7 and 14 show PC-hepc control. Lane 2 and Lane 9, Lane 3 and Lane 10, Lane 4 and Lane 11, Lane 5 and Lane 12, Lane 6 and Lane 13 show digestion products of PC-hepc by digestive enzyme for 0 s, 5 s, 2 min, 30 min, 60 min respectively.

Simulated digestion test is important for the oral safety assessment of genetically engineered proteins or peptides. If the proteins or peptides can be digested by the simulated gastric fluid or simulated intestinal fluid, they have less risk to cause allergy or toxicity. PC-hepc kept stable in simulated gastric fluid throughout the test of 60 min (Fig 1), indicating PC-hepc could hardly be digested in stomach. However, PC-hepc was digested in simulated intestinal fluid in less than 15 seconds (Fig 1). Thus PC-hepc is susceptible to intestinal digestion.

### PC-hepc had no adverse effects on general health, but caused increase of blood glucose in female rats

**Fig 2:**
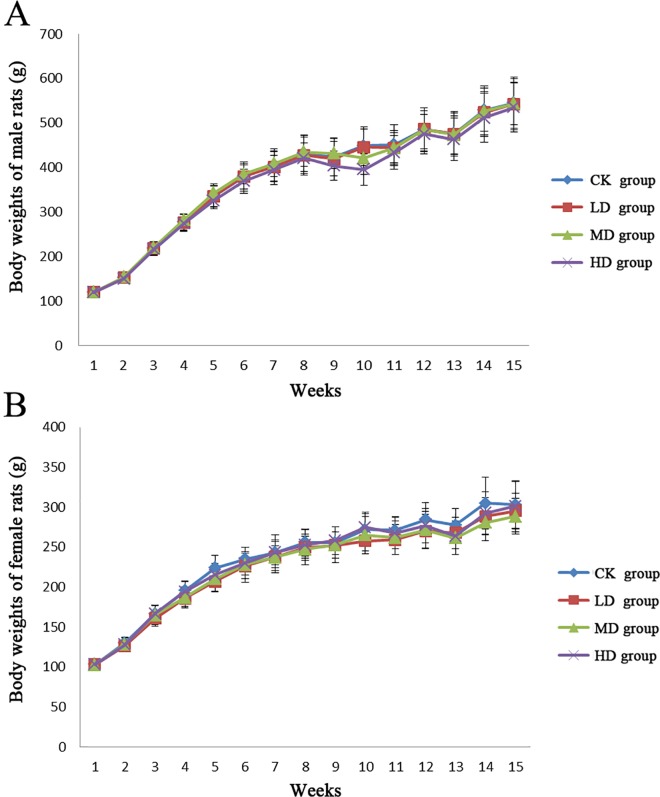
The body weights of the male and female rats during the experiment.

PC-hepc was conducted to male and female rats by formulation into feeds in different concentration. Throughout the study, no adverse effects were observed in both control group and trial groups (LD, MD, and HD group). Body weights of rats in control group and trial groups showed normal and similar growth patterns with no significant difference during the study (Fig 2). The total food consumption and food utilization of each group showed no significant difference in males (Table 1). However in females, the total food consumption of HD group showed a decreased tendency compared with CK group (*p*>0.05), which resulted in significant increase in food utilization of HD group (*p*<0.05).

The results of terminal hematology are shown in Table 2. No significant difference was observed in all indexes among female rats, while in male rats, three indexes showed significant differences (*p*<0.05) compared with CK group, including significant decrease of white blood cell (WBC) counts in the LD group, significant increase of hemoglobin (Hb) in LD group, and significant decrease of red blood cell distribution width (RDW) in MD group. However, these changes were within the normal ranges [23] and were not observed in HD group. Thus, these significant differences couldn’t indicate any adverse effects of PC-hepc on the hematology of rats.

The serum chemistry data are shown in Table 3. Glucose (GLU) concentration in all the female trial groups was significant increased (*p*<0.05) compared with CK group. Some other significant differences were observed in LD or MD group, including albumin (ALB) and alkaline phosphatase (ALP) in male rats and phosphorus (P), cholesterol (CHO), total phosphorus (TP) in female rats. All these changes were within the normal ranges [23].

### High concentration of PC-hepc caused the intestinal inflammation in female rats

The effects of PC-hepc on the main organs of rats were detected by organ/body weight ratio and histopathological examination. As shown in Table 4, there was no significant difference in organ/body weight ratio between control group and trial groups except a slight but significant increase of kidneys/body weight ratio in female HD group (*p*<0.05). Histopathological examination showed that there was more appearance of protein or even urinary cylinder in the kidney tubules of deep cortex and shallow medulla kidney of female HD group compared with female CK group. Histopathological examination also showed obvious increase of inflammation in the jejunum of female HD group compared with that of female CK group, however the jejunum of female LD group and female MD group was normal.

### Medium and high concentration of PC-hepc caused changes of gut microbiota in female rats

As PC-hepc caused the jejunum inflammation in female HD rats, which indicated high concentration of PC-hepc could have effects on the intestinal health in female rats, the gut microbiota of female rats was detected for further exploration. High throughput sequencing of 16S rRNA of gut microbiota from 48 female rats (four groups, six samples per group, and two time points, *i*.*e*. day 0 and day 90) produced 1.542 million clean reads, with 32,129 ± 1,852 (means ± s.d.) in average.

The Simpson diversity index was calculated to evaluate the effect of PC-hepc on the diversity of gut microbiota. No significant difference was found between female CK group and each female trial group both at day 0 and day 90 (S1 Fig). Besides, the total bacteria counts were measured by real-time PCR, and the results showed also no significant difference comparing female CK group with each female trial group (S2 Fig). Thus PC-hepc didn’t affect the total bacteria counts and diversity of gut microbiota in female rats.

Analysis of similarity (ANOSIM) was performed to assess the shifts of community structure of gut microbiota. The results indicated that the community structures of gut microbiota of all the female groups showed no significant difference on day 0. With the growth of rats, their gut microbiota shifted in all the groups including CK. The changes of gut microbiota with the growth of host are normal phenomenon. However, on day 90, the community structure of gut microbiota of female MD and HD groups was significantly different from that of female CK group (*p*<0.05), while the female LD group showed similarity with female CK group in gut microbiota (*p*>0.05). These results indicated that medium and high concentration of PC-hepc, or their digestive products, could cause changes of gut microbiota in female rats.

**Fig 3:**
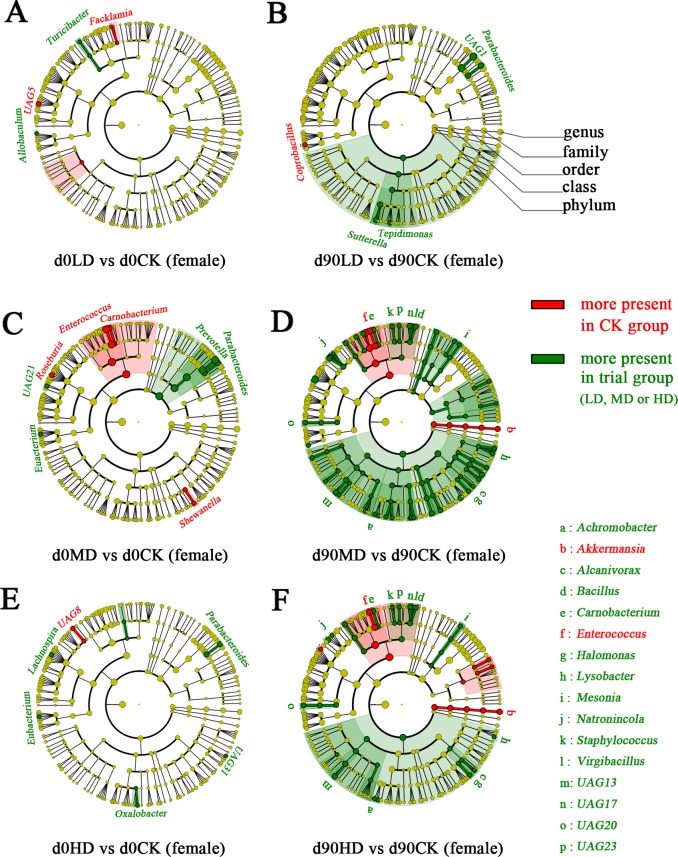
The bacterial genera affected by PC-hepc in gut microbiota of female rats according to LEfSe analysis. A, C, and E show the comparisons of gut microbiota between LD and CK, MD and CK, HD and CK on day 0 respectively. B, D, and F show the comparisons of gut microbiota between LD and CK, MD and CK, HD and CK on day 90 respectively. The circles from the outside to the inside indicate genus, family, order, class, phylum, and kingdom. Red indicates this taxon was more present in CK group than in trial group. Green indicates this taxon was more present in trial group than in CK group. In A, B, C, and E, all the differently present genera are coloured and labeled, while in D and F, all the differently present genera are coloured, but only the genera changed both in female MD and HD group on day 90 are labeled due to the space limitation in this figure.

To find out the bacterial genera that were affected by medium and high concentration of PC-hepc in gut microbiota of female rats, LEfSe was performed between each trial group and CK group. In accordance with the results of ANOSIM, very few genera were found to show difference between trial groups and CK group on day 0 (Fig 3A, 3C and 3E). On day 90, five genera in female LD group, forty-three genera in female MD group, and twenty-two genera in female HD group were found to be increased or decreased compared with female CK group (Fig 3B, 3D and 3F), of which sixteen genera showed the same change tendency in female MD and HD groups. Unexpectedly, although the *in vitro* study of PC-hepc showed inhibition effects on the growth of a variety of microorganisms [14], the LEfSe results indicated high concentration of PC-hepc, or maybe its digestive products, showed promotion effects on many kinds of gut bacteria. Only two genera, *Akkermansia* and *Enterococcus*, were presented less in both female MD and HD group than in female CK group.

### High concentration of PC-hepc caused different change patterns of gut microbiota in female and male rats

**Fig 4:**
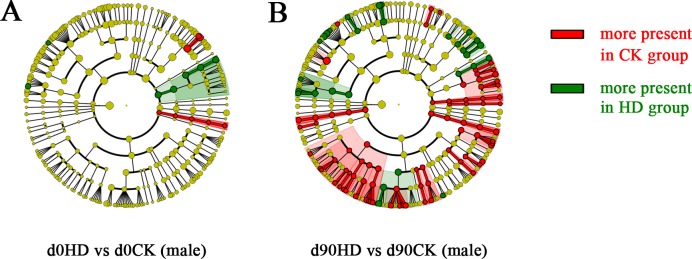
The bacterial genera affected by PC-hepc in gut microbiota of male rats according to LEfSe analysis. A shows the comparison of gut microbiota between male HD group and CK group on day 0, and B shows this comparison on day 90. The circles from the outside to the inside indicate genus, family, order, class, phylum, and kingdom. Red indicates this taxon was more present in CK group than in trial group. Green indicates this taxon was more present in trial group than in CK group.

The gut microbiota of male HD group rats were also sequenced and analyzed to compare with those of female HD group rats. The 16S rRNA sequencing of gut microbiota of male HD and CK group rats at day 0 and day 90 produced in average 94,732 ± 41,053 (means ± s.d., 6 sample per group) clean reads. The total bacteria counts and diversity of gut microbiota were also not affected by high concentration of PC-hepc in male rats. As revealed by the results of LEfSe analysis, the gut microbiota of male HD group and male CK group were very similar at day 0, but showed a large number of differential genera at day 90 (Fig 4). Different from the changing pattern of gut microbiota in female HD group which showed primarily bacteria promotion effects, among the 53 differentially present genera in male HD group, 37 of them were decreased compared with their relative abundance in CK group. Only the decrease of genus *Aeromicrobium* and *Anaerostipes* appeared in both male and female HD group compared with their respective CK group.

### Medium and High concentration of PC-hepc caused significant increase of fecal nitroreductase activity in female rats

**Fig 5:**
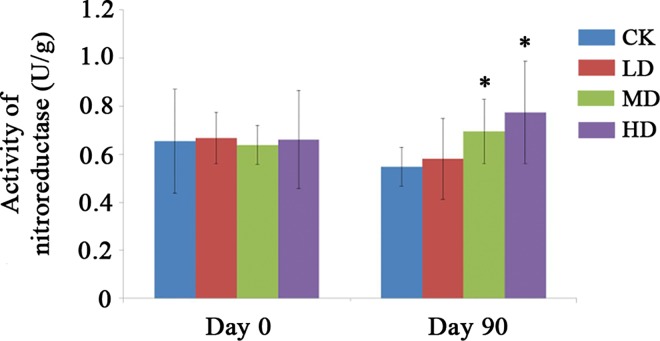
Activity of nitroreductase in the fecal samples of each group on day 0 and day 90. The superscript * indicates significant difference compared with CK group (*p*<0.05).

Three kinds of short-chain fatty acid (acetic acid, propionic acid and butyric acid) and four kinds of fecal enzymes (β-glucuronidase, β-glucosidase, nitroreductase, and β-galactosidase) were measured to determine the effects of PC-hepc on metabolism of gut microbiota, and the expressions of ZO1 and Occludin in jejunum were measured to determine the effects of PC-hepc on intestinal permeability. The results of the above tests showed no significant difference between the CK group and HD group (S3 Fig; S4 Fig; S5 Fig) except the significant decrease of concentration of acetic acid in male HD group (S3 Fig) and the significant increase of nitroreductase in female HD group (S3 Fig). As the increase of nitroreductase in female intestine might be related to the intestinal inflammation caused by PC-hepc, we further detected the concentration of nitroreductase in other trial groups of female rats. The results (Fig 5) showed on day 0 all the groups of female rats showed the similar concentration of nitroreductase, however on day 90, the concentration of nitroreductase in trial group increased in a dose-dependent manner, and both female MD group and HD group increase significantly (*p*<0.05) compared with female CK group.

### Digestion by SIF attenuated the inhibition effects of PC-hepc on gut microbial strains *in vitro*

**Fig 6:**
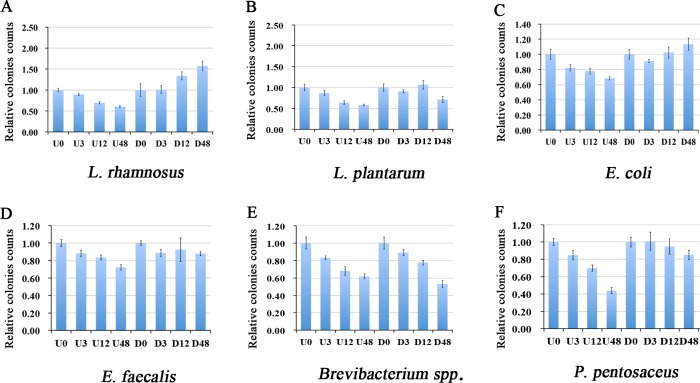
*In vitro* inhibition assay of undigested and digested PC-hepc on gut microbial strains. U0, U3, U12, and U48 indicated 0 μM, 3 μM, 12μM, and 48 μM undigested PC-hepc and their colonies counts were normalized by the average value of U0 (PBS buffer control); D0, D3, D12, and D48 indicated 0 μM, 3 μM, 12μM, and 48 μM digested PC-hepc. and their colonies counts were normalized by the average value of D0 (simulated intestinal fluid control). Each assay was repeated three times independently.

The in vitro effects of undigested and digested PC-hepc on gut microbial strains were determined using culture-based method. The results (Fig 6) showed that undigested PC-hepc could inhibit the growth of all the six microbial strains involved in this test assay in a dose-dependent manner. However, after digestion of PC-hepc by SIF, the inhibition effects were attenuated for most strains except *Brevibacterium* spp.. For *L*. *rhamnosus* and *E*. *coli*, the growth of bacteria was promoted with the increase of digested PC-hepc concentration. For *E*. *faecalis* and *P*. *pentosaceus*, the inhibition effects were lessened. The inhibition effect of 48 μM digested PC-hepc was similar to that of 3 μM undigested PC-hepc. For *L*. *plantarum*, the digested PC-hepc showed significant inhibition effects at 48 μM, but not at 12 μM. Thus it could be supposed that in most cases SIF digested PC-hepc has much less inhibition effects on gut microbial strains compared with complete PC-hepc.

## Discussion

A previous study showed that *Pichia pastoris*-expressed PC-hepc has a wide spectrum of *in vitro* antimicrobial activity, including *Micrococcus luteus*, *Corynebacterium glutamicum*, *Pseudomonas fluorescens*, and *Pseudomonas stutzeri* [14], indicating its promising application as antimicrobial agent, and also indicating the probability that PC-hepc could affect the bacteria in gut microbiota. As a kind of alternative to antibiotics as well as genetically engineered product, the oral safety assessment and evaluation of *in vivo* effects on gut microbiota of PC-hepc are essential. The pathogen infection animal models were not involved in this study, because we didn’t intend to prove the *in vivo* antimicrobial activities of hepcidin. In fact, as an antibacterial peptide, hepcidin may not be a good agent to treat *in vivo* pathogen infections, as our results showed that hepcidin could be digested by intestinal fluid. However, when used as food or feed bactericidal additive, this digestion characteristic of hepcidin could be a good advantage to avoid their impacts on gut microbiota of human or animals.

In our results, high concentration of PC-hepc affected the community structures of gut microbiota in both male and female animals with a sex-depend manner. The sex-depend changing pattern of gut microbiota is not an infrequent phenomenon. Bolnick et al. have systematically studied and discussed this phenomenon in different kinds of vertebrate including human and mice [24]. As they said, in both humans and mice, diet has previously been shown to modify the gut microbiota, but the possibility of sex and diet interactions has been overlooked. The same kind of orally conducted material can cause different changes in gut microbiota of male and female rats [25], and even the same kind of orally conducted bacterial strains can cause different changes in the physiology and metabolism of male and female host [26, 27].

We hypothesize that the difference of gut microbiota changing pattern in male and female rats might be due to the difference of digestive efficiency on PC-hepc. The PC-hepc peptides could not be digested in simulated gastric fluid but could be digested in simulated intestinal fluid. Thus PC-hepc peptides could get through the stomach without digestion and enter the intestinal tract. In the intestinal tract, the PC-hepc peptides began to be digested gradually, thus both PC-hepc peptides and their digestive products were present in the intestinal tract. We conjecture that the digestive products of PC-hepc, which would not act as antimicrobial peptides, could promote the growth of bacteria in gut by supplement of more amino acids. This conjecture could be partly proved by our *in vitro* inhibition assay of undigested and digested PC-hepc on gut microbial strains, as the SIF digested PC-hepc could promote the growth of *L*. *rhamnosus* and *E*. *coli*. Female rats have more digestive efficiency on PC-hepc, thus have more digestive products of PC-hepc in the gut; while male rats have less digestive efficiency, and thus have more PC-hepc in the gut. These could be the explanation of why most genera of gut microbiota increase in female rats and decrease in male rats. In low PC-hepc concentration group, the concentrations of both PC-hepc peptides and their digestive products were low, so the effects were not significant.

The intestinal inflammation and the change of gut microbiota observed in female HD group might have some relationships. The genus *Akkermansia*, a member of the Verrucomicrobia phylum, was found to decrease in female HD group. *Akkermansia muciniphila* has been reported to be an important gut bacterial symbiont, benefiting the host by its ability to colonize mucus and produce acetate and propionate [28]. The genus *Akkermansia* or *Akkermansia muciniphila* has been found to decrease in inflammatory bowel disease patients [29], obese individuals [30] and diet induced obese and type 2 diabetic mice [31], and increase in polyphenols [32] or metformin [33] treated mice. Most interestingly, metformin treatment to high-fat diet fed mice resulted in not only increase of *Akkermansia* but also improvement of glucose tolerance (reducing the area under the curve obtained after the glucose tolerance test), and administration of *Akkermansia muciniphila* could also improve the tolerance of glucose [33]. Thus the intestinal inflammation found in female HD group and the increase of blood glucose in all female groups might be intermediated by the decrease of *Akkermansia* in gut microbiota.

The increase of fecal nitroreductase activity in female rats might result from the change of gut microbiota as well. Nitroreductase is one of the procarcinogenic enzymes and the gut microbiota represents an enormous potential of bacterial nitroreductase activity [34]. The increase of nitroreductase results in more transformation of nitro-aromatic compounds to aromatic amines, which are harmful to the health of animals or human beings.

## Conclusion

In conclusion, the results showed PC-hepc could be digested in simulated intestinal fluid but not in simulated gastric fluid. PC-hepc had no adverse effects on general health, except causing increase of blood glucose (still in the normal value range of this index) in all trial groups of female rats and intestinal inflammation in HD group of female rats. Community composition of gut microbiota of female MD and HD groups shifted compared with control group, of which the decrease of genus *Akkermansia* might be related to the increase of blood glucose and intestinal inflammation according to previous reports about this genus. Besides, PC-hepc also caused significant increase of fecal nitroreductase activity in female MD and HD rats. High concentration of PC-hepc caused different changing patterns of gut microbiota in male and female rats, the reason of which remains unknown. Thus, our results suggest the uses of exogenous PC-hepc in normal dosage are safe, however excess dosage of it may cause intestinal disorder of animals.

## Supporting Information

S1 Fig The Simpson diversity index of gut microbiota of each group on day 0 and day 90 calculated according to the 16S rRNA sequencing data. (PDF)

S2 Fig The total bacteria counts of gut microbiota of each group on day 0 and day 90. (PDF)

S3 Fig The concentration of short-chain fatty acid (acetic acid, propionic acid and butyric acid) in the fecal samples on day 0 and day 90. The superscript * indicates significant difference compared with CK group (*p*<0.05).
(PDF)

S4 Fig The activities of four fecal enzymes (β-glucuronidase, β-glucosidase, nitroreductase, and β-galactosidase) in the fecal samples on day 0 and day 90. The superscript * indicates significant difference compared with CK group (*p*<0.05).
(PDF)

S5 Fig The expression of two tight junction proteins (ZO1 and Occludin) in jejunum. (PDF)
