# Supplementary material for: In Vivo Effects of Pichia Pastoris-Expressed Antimicrobial Peptide Hepcidin on the Community Composition and Metabolism Gut Microbiota of Rats
Source: PLoS One. 2016 Oct 21;11(10):e0164771. doi: 10.1371/journal.pone.0164771 (PMC5074506; doi:10.1371/journal.pone.0164771)

Supplementary Information

Fig. S2 The total bacteria counts of gut microbiota of each group on day 0 and day 90.

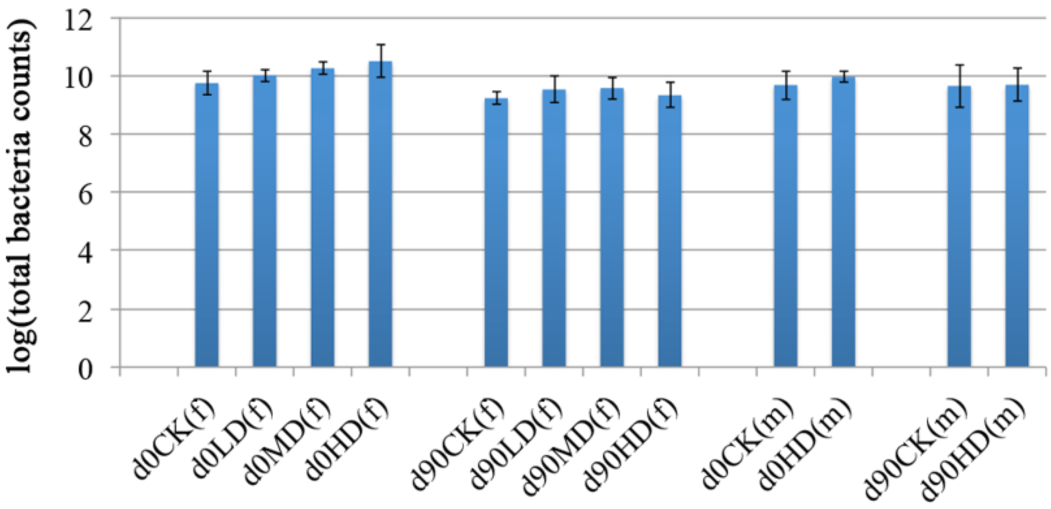

Supplement: S2 Fig — (PDF) [file pone.0164771.s002.pdf]
