# Supplementary material for: In Vivo Effects of Pichia Pastoris-Expressed Antimicrobial Peptide Hepcidin on the Community Composition and Metabolism Gut Microbiota of Rats
Source: PLoS One. 2016 Oct 21;11(10):e0164771. doi: 10.1371/journal.pone.0164771 (PMC5074506; doi:10.1371/journal.pone.0164771)

Supplementary Information

Fig. S3 The concentration of short-chain fatty acid (acetic acid, propionic acid and butyric acid) in the fecal samples on day 0 and day 90. The superscript * indicates significant difference compared with CK group (p<0.05).

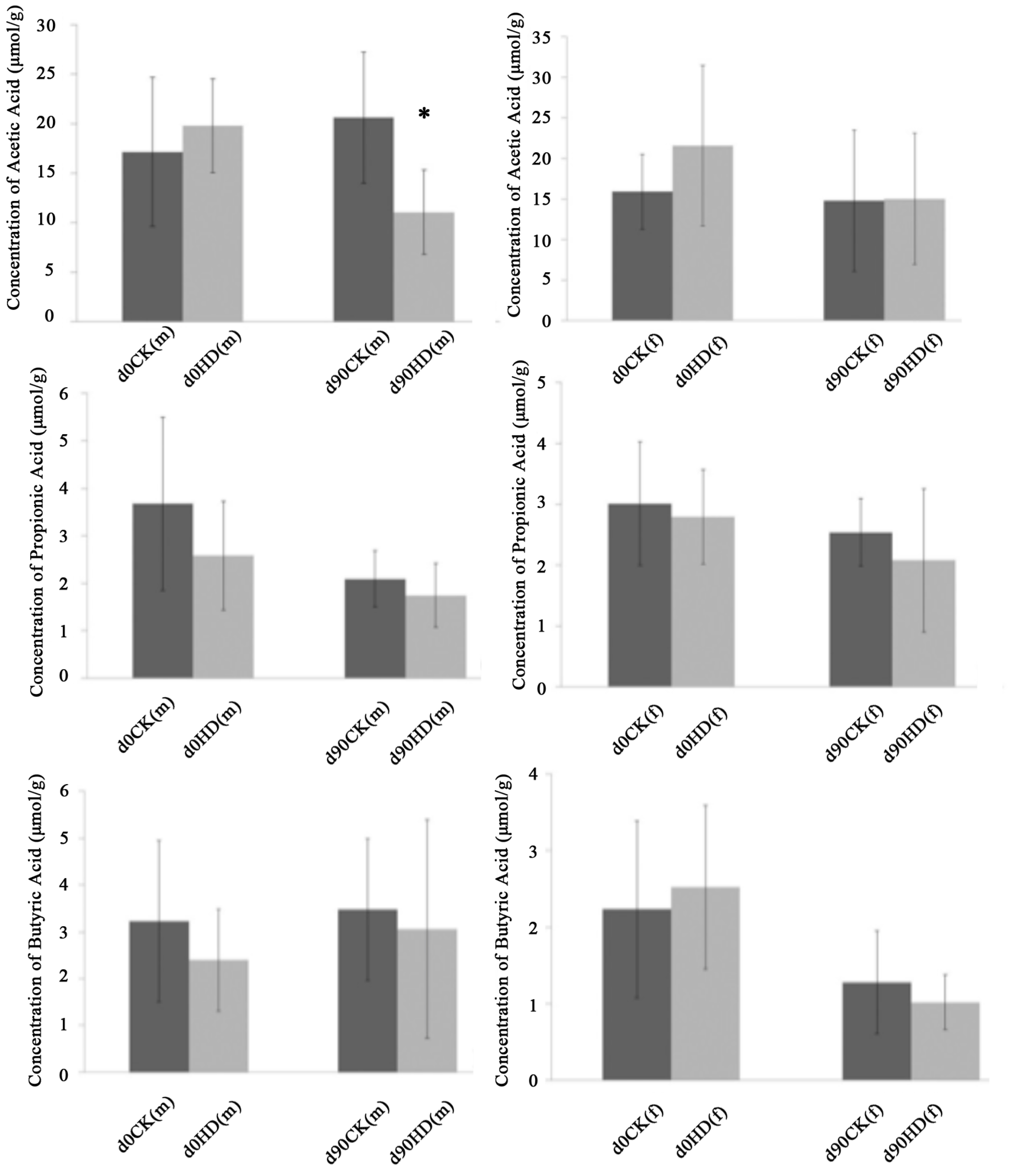

Supplement: S3 Fig — The superscript * indicates significant difference compared with CK group (p<0.05). (PDF) [file pone.0164771.s003.pdf]
