# Supplementary material for: In Vivo Effects of Pichia Pastoris-Expressed Antimicrobial Peptide Hepcidin on the Community Composition and Metabolism Gut Microbiota of Rats
Source: PLoS One. 2016 Oct 21;11(10):e0164771. doi: 10.1371/journal.pone.0164771 (PMC5074506; doi:10.1371/journal.pone.0164771)

Supplementary Information

Fig. S4 The activities of four fecal enzymes (β-glucuronidase, β-glucosidase, nitroreductase, and β-galactosidase) in the fecal samples on day 0 and day 90. The superscript * indicates significant difference compared with CK group (p<0.05).

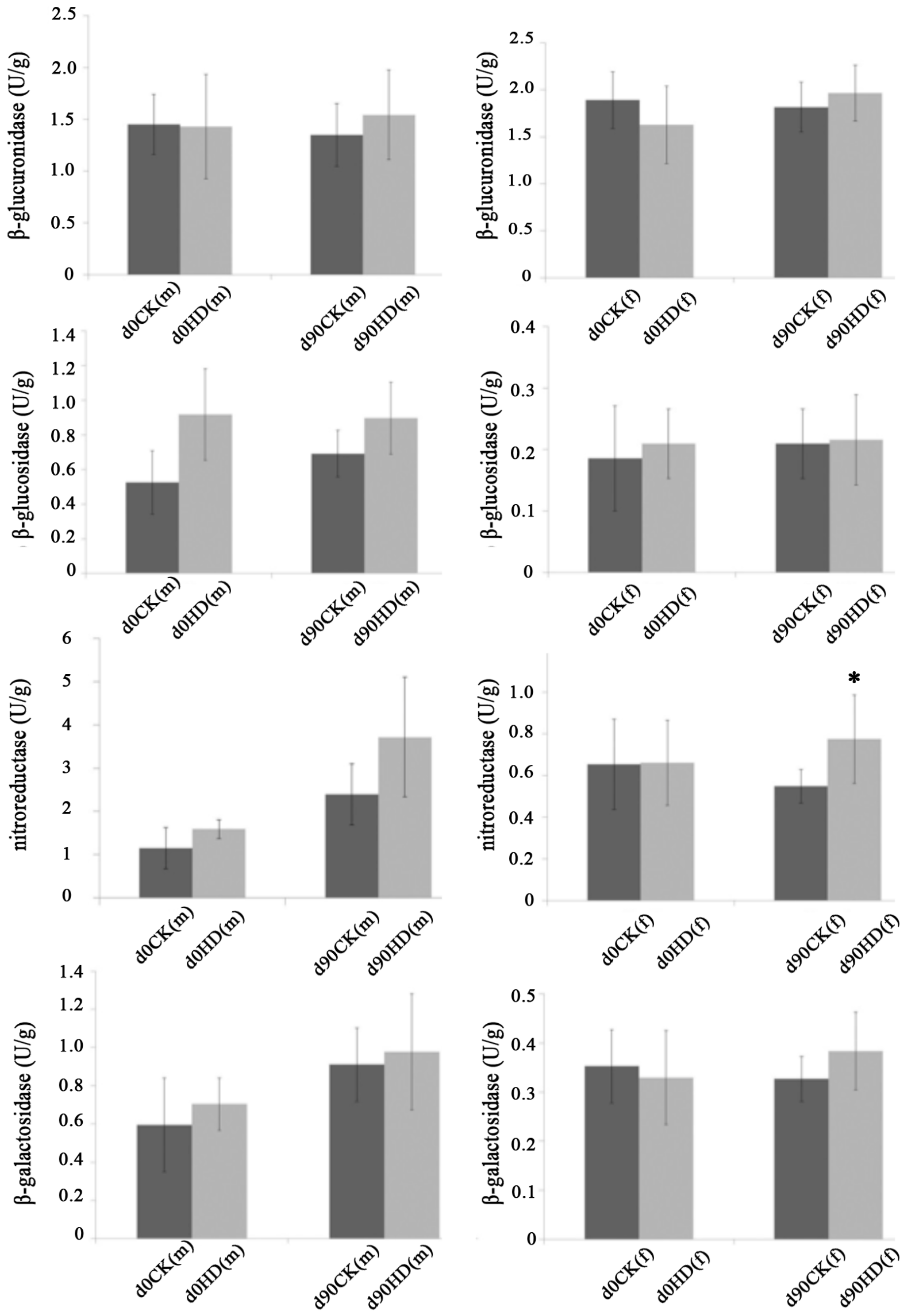

Supplement: S4 Fig — The superscript * indicates significant difference compared with CK group (p<0.05). (PDF) [file pone.0164771.s004.pdf]
