# Supplementary material for: In Vivo Effects of Pichia Pastoris-Expressed Antimicrobial Peptide Hepcidin on the Community Composition and Metabolism Gut Microbiota of Rats
Source: PLoS One. 2016 Oct 21;11(10):e0164771. doi: 10.1371/journal.pone.0164771 (PMC5074506; doi:10.1371/journal.pone.0164771)

Supplementary Information

Fig. S5 The expression of two tight junction proteins (ZO1 and Occludin) in jejunum.

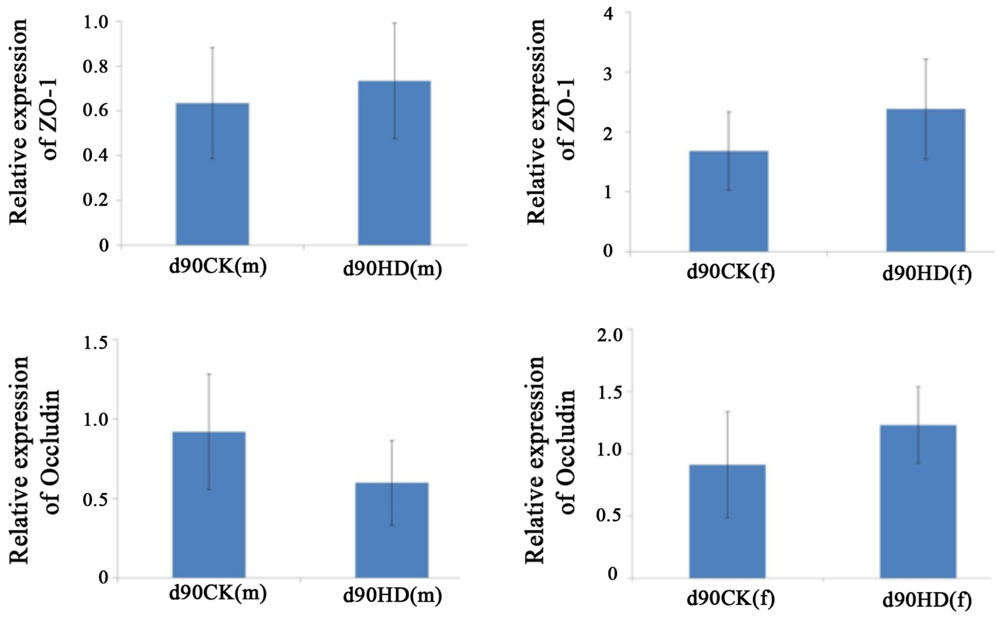

Supplement: S5 Fig — (PDF) [file pone.0164771.s005.pdf]
